# Short-term motor learning through non-immersive virtual reality task in individuals with down syndrome

**DOI:** 10.1186/s12883-017-0852-z

**Published:** 2017-04-14

**Authors:** Carlos Bandeira de Mello Monteiro, Talita Dias da Silva, Luiz Carlos de Abreu, Felipe Fregni, Luciano Vieira de Araujo, Fernando Henrique Inocêncio Borba Ferreira, Claudio Leone

**Affiliations:** 1 School of Arts, Sciences and Humanities, University of São Paulo, Av. Arlindo Béttio, 1000, Ermelino Matarazzo, São Paulo, 03828-000 Brazil; 2 School of Public Health, University of São Paulo, São Paulo, Brazil; 3 Harvard School of Public Health, Harvard University, Boston, MA USA; 4 Center for Neurosciences (NEC), University of São Paulo, São Paulo, Brazil; 5 Spaulding Rehabilitation Hospital and Massachusetts General Hospital, Harvard Medical School, Boston, MA USA

**Keywords:** Down syndrome, Virtual reality, Rehabilitation, User-computer interface, Physical therapy modalities

## Abstract

**Background:**

Down syndrome (DS) has unique physical, motor and cognitive characteristics. Despite cognitive and motor difficulties, there is a possibility of intervention based on the knowledge of motor learning. However, it is important to study the motor learning process in individuals with DS during a virtual reality task to justify the use of virtual reality to organize intervention programs. The aim of this study was to analyze the motor learning process in individuals with DS during a virtual reality task.

**Methods:**

A total of 40 individuals participated in this study, 20 of whom had DS (24 males and 8 females, mean age of 19 years, ranging between 14 and 30 yrs.) and 20 typically developing individuals (TD) who were matched by age and gender to the individuals with DS. To examine this issue, we used software that uses 3D images and reproduced a coincidence-timing task.

**Results:**

The results showed that all individuals improved performance in the virtual task, but the individuals with DS that started the task with worse performance showed higher difference from the beginning. Besides that, they were able to retain and transfer the performance with increase of speed of the task.

**Conclusion:**

Individuals with DS are able to learn movements from virtual tasks, even though the movement time was higher compared to the TD individuals. The results showed that individuals with DS who started with low performance improved coincidence- timing task with virtual objects, but were less accurate than typically developing individuals.

**Trial registration:**

ClinicalTrials.gov Identifier: NCT02719600.

## Background

Down syndrome (DS) is typically characterized by an additional chromosome, or trisomy 21 (Hsa21), with incidence of 1 in 750 live births and is considered one of the most frequent cause of learning difficulties [1].

The alterations made by individuals with DS may pose difficulties in functionality and independence to perform activities of daily living even during adulthood. Mancini et al. [2] reported that individuals with DS have less functional performance than individuals without motor and cognitive dysfunction with alterations in cognitive, motor areas and social function.

Due to the importance of learning motor skills for individuals with DS, it is important that practitioners use the knowledge derived from motor learning, which will enable an intervention program based on scientific evidence from this area of expertise. Examining how motor skill learning can be enhanced in individuals with DS is not only of theoretical interest, but may also have important practical implications for the lives of those affected by DS [3].

Some studies about motor learning in DS [3–6] were found, as well as a study about how individuals perform in a timing coincident task in a real environment using Bassin Anticipatory timing and the results showed that it is a good tool to evaluate improvement on motor performance in people with DS [7] but little was found on motor learning in a virtual reality task in individuals with DS [8]. Recently, with the growing accessibility of computer-assisted technology, rehabilitation programs increasingly use virtual reality environments to enhance dedicated practice, thus, to study the use of virtual reality becomes essential [9–12]. Also, the use of the real task (Bassin Anticipatory timing) present some difficulties to be used worldwide considering its size, weight and price, in addition if someone needs to import the equipment, they must anticipate going through bureaucratic red tape, a long waiting time and difficulty for technical support. Thus, it is important to verify the performance of DS individuals in a virtual task using a computer that can offer visual and auditory feedback and facilitate the use of a coincident timing assessment for rehabilitation professionals to predict the anticipatory timing. Virtual task with 3D images has been used in different devices and represent the future in technology promoting independence and functionality in the daily life tasks for individuals with DS.

Given this background, the aim of the current study was to assess the motor learning capacity of individuals with DS in a non-immersive virtual reality task. Based upon the above deliberations, we hypothesized that individuals with DS will improve their coincident time of movement during acquisition in a 3D task, in addition to maintaining performance for the retention phase and transfer the performance with an increase of speed, however with lower performance and more variability when compared to the control group.

## Methods

### Participants

A total of 40 individuals participated in this study, 20 of whom had DS (11 males and 9 females, mean age = 19 years, ranging between 14 and 30 yrs.) and 20 typically developing individuals (TD) who were matched by age and gender to the individuals with DS. Criteria for inclusion were a medical diagnosis of DS and the ability to understand the task. The ability to understand the task was: after two demonstrations from the experimenter (one showing right reach and other showing wrong reach) the participant had two attempts to show if he/she understood the task. All participants were from school program and alphabetized. Exclusion criteria were disorders in cognitive function that would prevent comprehension of the experimental instruction.

### Material and apparatus

This study used software that uses 3D images to reproduce a coincidence-timing task developed and tested by the Department of Electronic System Engineering of the Polytechnic School, University of São Paulo (EP/USP) (see also Silva et al. [13] and de Mello Monteiro et al. [14]) and upgraded by the Laboratory of Information Systems from the University of São Paulo.

This software enabled the coincidence-timing task to be done by pressing the space key on a keyboard. The coincident timing task was based on the Bassin Anticipation Timer [7, 15–21] and consisted of a task used worldwide by many authors (see Chiviacowsky et al. [3]; de Mello Monteiro et al. [14]; Corrêa et al. [18]) aiming to assess and verify motor learning considering the performance obtained by decreasing the errors, or the variability of errors.

**Fig. 1:**
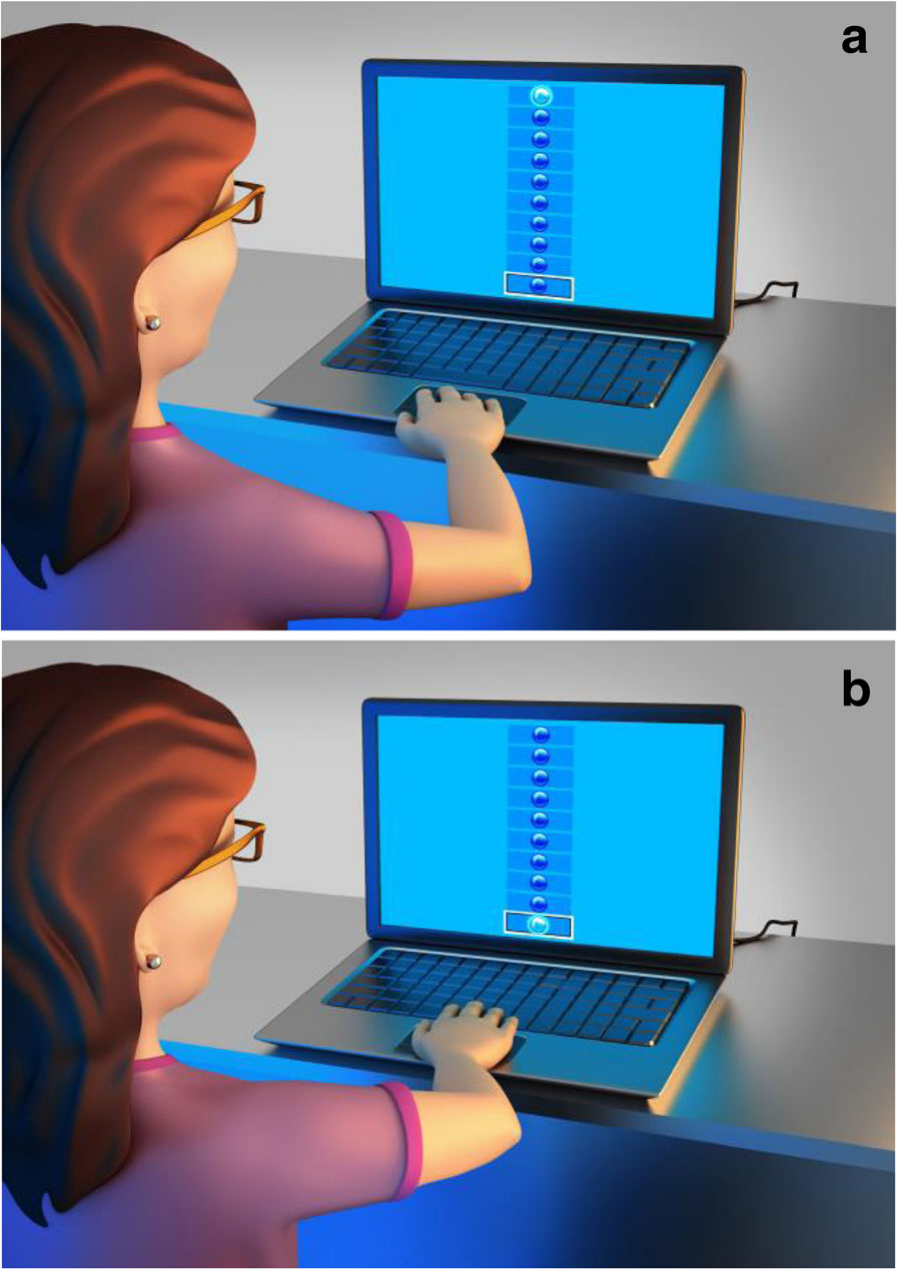
Coincident timing task using 3D image: initial position (**a**) and final position **b**

To this end, 10 3D–cubes were displayed simultaneously in a vertical column on a monitor. The cubes created turned on (i.e., changed from blue to green) and off sequentially (from top to bottom) until the target cube (i.e., the tenth cube) was reached. The task for the participant was to press the space bar on the keyboard in the exact time to hitting the target object (Fig. 1).

### Procedure and design

Participants performed the task individually in a quiet room with only the researcher who gave the instructions to the individual present. The computer was placed on a table. The participants were seated in chair, which was adjusted in height according to the needs of the individual. Also a footrest was available, if required. After being seated, the experimenter explained the task verbally equally for all participants and as mentioned before gave two demonstrations of how to perform the coincidence-timing tasks and two trials as tests for the participant, as a way to access the comprehension of the task. These attempts did not count for the experiment. The participants were instructed to place the preferred hand on a mark in front of the target (the location was individually adjusted but ranged from 2 to 4 cm from the target keyboard). Once the first top cube turned on, the individual had to move his or her hand to either touch the target key on the keyboard, exactly at the moment coinciding with the bottom target cube turning on. During the tasks, the experimenter did not provide any encouragement or reward. Different sounds were provided as feedback for a hit or miss during acquisition, retention and transfer, the range of error being −200 to 200 ms. We chose auditory feedback because it is viable and better differentiated by the computer, and is described as a widely used form with evidence of effectiveness as sensorial feedback [22].

The primary purpose of practice is considered not only to facilitate the performance of temporary effects during acquisition but also provide improved durable performance (assigned to the learning) of the retention and transfer tests. To this end, “Retention test” was used as a way to measure the capacity to maintain the same performance acquired with the practice after a period with no contact with the task and “Transfer test” was used to assess the capacity to maintain the same performance acquired with practice when changing something in the task; it evaluates the ability to transfer the performance in a similar task or environment.

During the three phases of the study, we used blocks of 5 attempts each to ensure fairness in the evaluation of performances of the participant, as used in several studies [3, 14, 18]. All participants made 20 trial acquisitions (divided into 4 blocks), 5 trial retentions after 10 min with no contact with the task (1 block) and 5 trial transfers immediately after retention trials (1 block). Thus, each participant had 32 trials (2 test trials – that did not count for the study; 20 acquisition trials; 5 retention trials; 5 transfer trials). During acquisition and retention trials, the cubes simulated a dropped light movement with the turning on and off of the lights in an interval of 400 ms between positions change, while during transfer light movement was increased with 300 ms between positions.

### Data analysis

The dependent variable used was the timing error, i.e., constant error (CE), absolute error (AE) and variable error (VE).

#### Constant error (CE)

The calculation of CE was done by the simple arithmetic average of the error values, considering the algebraic sign (negative or positive) in a series of attempts (block of trials). It represents the direction of error if it is late or early.

$$ CE=\left[\Sigma \left( Xi\hbox{--} T\right)/ N\right] $$

In which: Σ = sum, i = number of attempts, Xi – score of the trial i, T = distance from the target, N = number of trials.

#### Absolute error (AE)

The individual values of AE were used with no algebraic sign (all values are positive) before proceeding to the calculation of average. The absolute error represents the individual as well as combinations effects of tasks characteristics [23]. AE represents the accuracy measurement.

$$ \mathrm{AE}=\left[\Sigma\ \left(|\ \mathrm{Xi}\hbox{--} \mathrm{T}\right)/\mathrm{N}\right] $$

In which: Σ = sum, | = “absolute value of”, i = number of attempts, Xi – score of the trial i, T = distance from the target, N = number of trials.

#### Variable error (VE)

To calculate the variable error, we squared the difference between each score and the individual CE mean, then sum all and divided by the number of trials. Then we compute the square root of this number. It represents the variability of error.

$$ VE=\sqrt{\left[\sum {\left( Xi- CE\right)}^2/ N\right]} $$

In which: Σ = sum, i = number of attempts, Xi– score of the trial i, CE = constant error, N = number of trials.

The timing error was defined as the time difference between the moment the target cube switched on (arrival time) and the moment at which the keyboard was touched. The dependent variable for the CE was submitted to a 2 (group: DS and TD) by 2 (blocks) ANOVA, repeated measures were utilized for the last factor.

The cognitive and motor function in people with DS is very different within them, so we divided the groups in order to find out if the performance on the first block would be related to the comprehension of the task, affecting the performance. For AE and VE the groups were divided by two subgroups according to the performance in the first acquisition block (10 individuals with best movement time: Performance Group A and 10 individuals with worse movement time: Performance Group B) the dependent variables were submitted to a 2 (group: DS and TD), by 2 (performance groups: A and B) by 2 (blocks) ANOVA, repeated measures were utilized for the last factor. For the factor block separate comparisons were made for acquisition (first acquisition block A1 versus final acquisition block A4), retention (A4 versus retention block R) and transfer (A4 versus transfer block T). Post hoc comparisons were carried out using Tukey-HSD test (*p* < .05).

## Results

The t-test found no statistical difference for age between groups of best movement time and worst movement time for both DS-group (18.4 ± 5; 20.5 ± 3, *p* = 0.154, respectively) and TD-group (19.3 ± 4; 18.8 ± 4, *p* = 0.400, respectively).

### Acquisition

#### Constant error

**Fig. 2:**
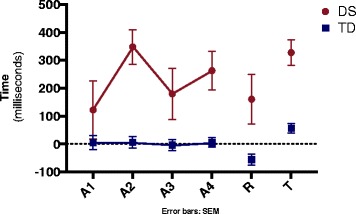
Constant error between groups on acquisition, retention and transfer blocks. A1-A4- refers to four acquisition blocks; R- refers to retention block; T- refers to tranfer block; DS- Down syndrome; TD- typical development; SEM- standard error of mean

Figure 2 present the CE’s during acquisition tasks among the DS- and TD-groups. The ANOVA did not reveal any effect or interaction for Block. However, a significant main effect for Group was found, F(1, 38) = 6.62, *p* = .014, ŋ^2^ = .15. It suggests that a difference in movement time between groups occurred, in other words DS-group had a much larger constant error (M = 193 ms) than TD-group (M = 6 ms) and in both groups the directional trend was late in movement.

#### Absolute error

**Fig. 3:**
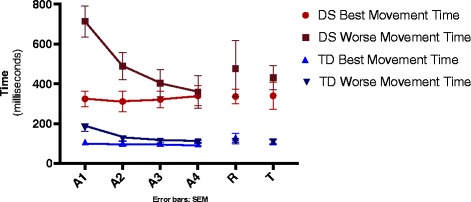
Absolute error between groups on acquisition, retention and transfer blocks. A1-A4- refers to four acquisition blocks; R- refers to retention block; T- refers to tranfer block; DS- Down syndrome; TD- typical development; SEM- standard error of mean

The pattern of absolute errors is illustrated in Fig. 3. Significant effects were found for Group F(1, 36) = 75.2, *p* < .001, ŋ^2^ = .68, Performance Group F(1, 36) = 11.7, *p* = .002, ŋ^2^ = .25 and Block, F(1, 36) = 12.4, *p* = .001, ŋ^2^ = .26, and interactions for Group by Performance Group F(1, 36) = 5.18, *p* = .029, ŋ^2^ = .13, Blocks by Performance Group F(1, 36) = 13.1, *p* = .001, ŋ^2^ = .27, Blocks by Group F(1, 36) = 4.66, *p* = .038, ŋ^2^ = .12 and Blocks by Performance Group by Group F(1, 36) = 6.65, *p* = .014, ŋ^2^ = .16. It indicates that DS- group had a much larger absolute error (M = 434 ms) than TD- group (M = 123 ms) and the Performance Group B has a much larger absolute error (M = 340 ms) than the Performance Group A (M = 217 ms). The post hoc test showed that this difference can be attributed to the DS-group that had a larger movement time in the Performance Group B (M = 536 ms) than in the Performance Group A (M = 332 ms), while for the TD-group this difference did not occurr (M = 143 ms versus 103 ms, respectively).

For Blocks, in the final block the movement time was smaller (M = 226 ms) when compared to the first block of acquisition (M = 331 ms) in both groups. The post hoc test showed that this difference can be attributed to the DS-group that showed worse performance in the first block of acquisition- Performance Group B (M = 714 ms) when compared to the final block of acquisition (M = 359 ms), for the other three groups this difference did not occurred.

#### Variable error

Similar to the absolute error, significant effects were found for Group F(1, 36) = 35.5, *p* < .001, ŋ^2^ = .50, Performance Group F(1, 36) = 35.4, *p* < .001, ŋ^2^ = .50 and Block, F(1, 36) = 33.0, *p* < .001, ŋ^2^ = .48, and interactions for Group by Performance Group F(1, 36) = 14.5, *p* = .001, ŋ^2^ = .29, Blocks by Group F(1, 36) = 20.6, *p* < .001, ŋ^2^ = .36, Blocks by Performance Group F(1, 36) = 14.1, *p* = .001, ŋ^2^ = .28, and Blocks by Performance Group by Group F(1, 36) = 8.64, *p* = .006, ŋ^2^ = .19. It indicates that DS- group had a much larger variable error (M = 107 ms) than TD- group (M = 39 ms) and the Performance Group B has a much larger variable error (M = 107 ms) than the Performance Group A (M = 39 ms). The post hoc test showed that this difference can be attributed to the DS-group that had a larger movement time -Performance Group B (M = 163 ms) than in the Performance Group A (M = 51 ms), while for the TD-group this difference did not occur (M = 51 versus 27 ms, respectively).

**Fig. 4:**
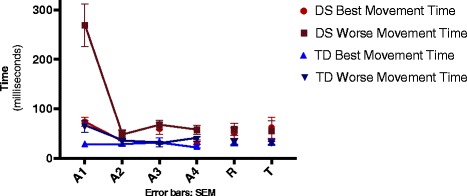
Variable error between groups on acquisition, retention and transfer blocks. A1-A4- refers to four acquisition blocks; R- refers to retention block; T- refers to tranfer block; DS- Down syndrome; TD- typical development; SEM- standard error of mean

For Blocks, in the final block of acquisition the movement time was smaller (M = 37 ms) when compared to the first block of acquisition (M = 109 ms) in both groups. The post hoc test showed that this difference can be attributed to the DS-group that showed worse performance in the first block of acquisition -Performance Group B (M = 268 ms) when compared to the final block of acquisition (M = 57 ms), for the other three groups this difference did not occurred (Fig. 4).

### Retention

There was significant effect of Block only for constant error, F(1, 38) = 5.05, *p* = .030, ŋ^2^ = .12. The result indicates that in the final acquisition block the constant error was much larger (M = 135 ms) than the retention block (M = 53 ms), in addition, the directional trend was late in movement. For the absolute and variable error the ANOVA did not find any main effects and interactions, suggesting that the patterns of movement were no different during retention as compared to final acquisition block for absolute (DS- 407 to 349 ms and TD- 119 to 102 ms, respectively) and variable errors (DS- 55 to 43 ms and TD- 33 to 31 ms, respectively).

### Transfer

Similarly to retention test, there was significant effect of Block only for constant error, F(1, 38) = 6.44, *p* = .015, ŋ^2^ = .15. This result indicates that in the transfer block the constant error was much larger (M = 135 ms) than the final acquisition block (M = 193 ms), in addition the directional trend was late in movement. For the absolute and variable error there were no significant effects of Block, suggesting that the patterns of movement were no different during transfer as compared to final acquisition block for absolute (DS- 385 to 349 ms and TD- 108 to 102 ms, respectively) and variable errors (DS- 58 to 43 ms and TD- 32 to 31 ms, respectively).

## Discussion

The main objective of this study was to determine whether individuals with DS can learn a non-immersive virtual task, and if there is the ability to adapt new environmental demands through improved performance in the acquisition and transfer with increasing speed. Therefore, the chosen task was coincident timing with (3D) virtual object held on a computer screen.

According to the results, the initial hypothesis of improved performance in a virtual task was partly confirmed, it was found that only Down Syndrome in Performance Group B showed significant improvement in performance in the execution of the task between the first block (M = 714 ms) and the final block of the acquisition (M = 359 ms). Latash [24] says that performance of individuals with DS shows dramatic improvement with practice even in tasks that are very simple, and seem to offer little room for improvement, but our results show that for the task used, this improvement in performance occurred only with individuals with low performance in the beginning.

Based on the present findings, we can only speculate why the individuals with DS with high performance did not improve. Considering that the motor and cognitive function in people with DS are very different among them [25] even between DS from school programs (all alphabetized), probably the task was easier for the group with high performance and they already show the best score from the beginning of the protocol. This speculation can be supported by Malak et al. [26], who evaluated the association of the motor and cognitive development in children with DS and concluded that the motor development is associated with cognitive development, i.e. mental age.

However, the primary purpose of practice is not only to facilitate the performance of temporary effects during acquisition but also provide improved durable performance (assigned to the learning) of the retention and transfer tests. From the results obtained, there was no significant difference between the last block of acquisition with retention and transfer blocks in absolute error in both the TD (102 ms to 119 ms, respectively) group, as in DS (349 ms to 407 ms, respectively) group. That is, both groups maintained the ability to perform the task after a certain time without running.

It is important to note that the small number of repetitions was sufficient to improve performance, but only in the group with DS who had more difficulty in the beginning of the task. For this group, even considering an acquisition with few repetitions, participants underwent an initial phase - characterized by a greater number of errors, inconsistencies and high demand attention - until a later stage, which is characterized by greater consistency, fewer errors and likely reduced demand attention. Those DS individuals organized their reaching and aiming movements to achieve the precision dictated by the task demands, while optimizing movement speed and energy efficiency. When faced with unexpected changes to the task demands, they are usually very adept at adjusting their movement trajectories to accommodate the new environmental constraints [27].

Considering the results of the last acquisition block (TD = 102 ms and DS = 349 ms) with transfer block (TD = 108 ms and DS = 385 ms), there were no statistically significant differences between blocks in both groups. Therefore, a situation with increased speed of the task did not cause significant performance drop. Again, primarily participants with DS who started with low performance after practice showed good adaptability to a new situation in coincident timing task. Considering the variable error, except for the first block of the group with DS with worse performance, the variability of error is similar between DS and people with typical development.

Those findings are partly in line with the notion that DS individuals can improve their ability in a virtual computer task (see Berg et al. [28]), The recent development in the area of motor control allows researchers and practitioners to tap into these reserves and use quantitative indices of changes in motor synergies with practice to optimize training programs for DS individuals [24].

Comparing the DS and TD groups, it was found that the DS-group had worse performance in acquisition than the group with typical development. This difference was also observed in the phases of retention and transfer. In DS, inability to effectively control movement through online processing of information may account for the slowness that is characteristic of their performance. They likely have difficulties due to less time in deceleration of reaching, less anticipatory capacity, longer movement times [29] and stability challenges of ligamentous laxity and hypotonia (see Galli et al. [30] and Cisterna et al. [31]).

Another factor that may contribute to greater difficulty in performance in participants with DS is the movement pattern and strategy used to accomplish the task. Individuals with DS have difficulty performing precision goal-directed movement with any degree of speed or efficiency and the movement performed took twice as long to complete than TD person [27] and individuals with DS have preference for patterns of muscle activation characterized by higher levels of co-contraction, that is, simultaneous activation of muscle pairs acting at a joint in opposite directions (“agonist-antagonist muscle pairs”) [32].

Considering the constant error and variable error, result indicates that in CE all individuals (DS and TD) had a trend to delay the movement. Masumoto et al. [33] evaluated adolescents with DS in a bimanual and unilateral tapping task and found also that they presented delay to reach the timing task. However in VE the DS- group had a much larger variable error (M = 107 ms) than TD- group (M = 39 ms), our findings confirm data from Torriani-Pasin et al. [7], even in a 3D task the individuals with Down syndrome tend to perform tasks in unstable and irregular coincidence of visual-motor systems, when compared to control subjects. Individuals with DS looked at experimental objects less frequently than their typically and children with typical development spent more time than DS in the highest visual stimulation complexity zone [34].

Another difficultly for DS individuals is in the deficit of the perceptual-motor abilities, which are responsible for the abilities that support the acquisition of several motor skills. Individuals with DS rely more on feedback control, whereas they have problems with movement planning and feed-forward control as showed by Kearney and Gentile [29] and Spanò et al. [35], the different strategy operated by individuals with DS leads to a different task performance. Probably DS has a deficit in voluntary motor commands and preprogramming of actions, which results in altered strategies of movement that enhance security and stability instead of efficiency of the performance [36].

## Conclusions

Based on the present findings, individuals with DS are able to learn movements from virtual tasks, even though the movement time was higher compared to the TD individuals. The results showed that individuals with DS who started with low performance improved coincidence- timing task with virtual objects, but were less accurate than typically developing individuals.

Although individuals with DS having different exploratory actions, perceptual-motor abilities, pattern and movement strategies, results showed the ability to improve performance in non-immersive virtual tasks especially for individuals who have more difficulties, which can encourage the use of virtual environments in programs of motor habilitation in individuals with DS.

### Limitations and future studies

As limitations of the study, we could not assess the cognitive function and mental age of the participants with DS or their access to computers or video games. This information could give more explanations about the difference between the groups with worse and better performance.

When compared to previous studies in this field, the present study included a rather heterogeneous population with a wide age range including adolescents and adults with different gender. Even though we cannot rule out the effects of these factors, it is believed that this did not affect the results to a great extent. Both groups were carefully matched for age and all participants in the DS group showed to be able to perform the task.

Moreover, the computer program we used limited the outcome parameters to non-immersive virtual task and we could not compare with a real task, probably transfer to a real task could have given even better insight into our findings. Therefore, for future studies it would be recommended to not only look at virtual task but also transfer for real environment since this could provide valuable information. Additionally, we suggest that this non-immersive task of virtual reality may be an option to be reproduced in others diseases with motor and cognitive disabilities.

## Abbreviations

AE: Absolute Error
CE: Constant Error
DS: Down Syndrome
TD: Typical Development
VE: Variable Error
